# Managing expectations: assessment of chemistry databases generated by automated extraction of chemical structures from patents

**DOI:** 10.1186/s13321-015-0097-z

**Published:** 2015-10-06

**Authors:** Stefan Senger, Luca Bartek, George Papadatos, Anna Gaulton

**Affiliations:** GlaxoSmithKline, Stevenage, Hertfordshire SG1 2NY UK; European Molecular Biology Laboratory - European Bioinformatics Institute (EMBL-EBI), Wellcome Trust Genome Campus, Hinxton, Cambridge CB10 1SD UK

**Keywords:** Patents, Patent chemistry databases, SureChEMBL, IBM SIIP

## Abstract

**Background:**

First public disclosure of new chemical entities often takes place in patents, which makes them an important source of information. However, with an ever increasing number of patent applications, manual processing and curation on such a large scale becomes even more challenging. An alternative approach better suited for this large corpus of documents is the automated extraction of chemical structures. A number of patent chemistry databases generated by using the latter approach are now available but little is known that can help to manage expectations when using them. This study aims to address this by comparing two such freely available sources, SureChEMBL and IBM SIIP (IBM Strategic Intellectual Property Insight Platform), with manually curated commercial databases.

**Results:**

When looking at the percentage of chemical structures successfully extracted from a set of patents, using SciFinder as our reference, 59 and 51 % were also found in our comparison in SureChEMBL and IBM SIIP, respectively. When performing this comparison with compounds as starting point, i.e. establishing if for a list of compounds the databases provide the links between chemical structures and patents they appear in, we obtained similar results. SureChEMBL and IBM SIIP found 62 and 59 %, respectively, of the compound-patent pairs obtained from Reaxys.

**Conclusions:**

In our comparison of automatically generated vs. manually curated patent chemistry databases, the former successfully provided approximately 60 % of links between chemical structure and patents. It needs to be stressed that only a very limited number of patents and compound-patent pairs were used for our comparison. Nevertheless, our results will hopefully help to manage expectations of users of patent chemistry databases of this type and provide a useful framework for more studies like ours as well as guide future developments of the workflows used for the automated extraction of chemical structures from patents. The challenges we have encountered whilst performing this study highlight that more needs to be done to make such assessments easier. Above all, more adequate, preferably open access to relevant ‘gold standards’ is required.

**Electronic supplementary material:**

The online version of this article (doi:10.1186/s13321-015-0097-z) contains supplementary material, which is available to authorized users.

## Background

In commercial Research and Development it is essential to seek protection by means of intellectual property laws for scientific results that are considered as being of value. To this end, the first public disclosure of new chemical entities often takes place in patent applications prior to publication in scientific journals. The average time lag between patents and the scientific literature for chemistry disclosures has been estimated to be in the order of 1–3 years [1, 2]. Importantly, only a small fraction of the results reported in patent applications will subsequently propagate to scientific journals. In fact, there is evidence that a large percentage of new science which appears in patents is never published elsewhere [3]. As a consequence, patents are an essential source of information for research scientists.

Traditionally, relevant content is manually extracted from patent documents, similar to the process applied to publications in scientific journals. However, this is a very costly approach and was consequently only available to scientists at a premium price from commercial vendors. Whilst the manual process, if done well, provides accurate information, compromises have to be made in regards to the coverage and turnaround time due to the sheer volume of published patent applications across the world. For pharmaceutical patents, for example, vendors might decide to focus on specific classes of proteins (e.g. kinases or GPCRs) and only extract information from patents that mention members of these target classes in either the title or abstract.

In recent years, workflows have been developed that allow for large scale chemical annotation content to be extracted in a batch automated fashion by means of text- and image-mining. One example for such a workflow is the Strategic IP Insight Platform (SIIP) from IBM [4]. At the end of 2011, IBM announced [5] that in collaboration with four drug companies it would provide to NIH (National Institutes of Health) a database that was generated using the IBM SIIP. The first version of the database that was provided contained more than 2.4 million chemical structures extracted from about 4.7 million patents, as well as 11 million biomedical journal abstracts from 1976 to 2000. For this period, images in patents were not processed and chemical structures were only derived from text. Subsequently, the coverage was extended up to the end of 2010 and chemical structures were derived from US Complex Work Units (CWU) [6] and images in WO patents for the period 2001–2010. Chemical structures generated using IBM SIIP are, for example, available in PubChem [7] and UniChem [8]. Two years later, EMBL-EBI announced [9] that chemistry extracted from full text patent documents that had been previously licensed by the commercial patent chemistry database SureChem would transfer into the public domain, and it is now freely available at http://www.surechembl.org. SureChEMBL contains searchable patent documents (applications and granted patents) from the USPTO, EPO and WIPO, (United States Patent and Trademark Office, European Patent Office and World Intellectual Property Organization, respectively) along with English abstracts of Japanese patents. Similarly to the IBM system, chemicals are extracted from full text (title, abstract, claims, description; from 1976 onwards), images (from 2007 onwards) and CWUs (for US patents only, from 2007 onwards) using an automated pipeline of chemical entity recognition, name-to-structure and image-to-structure tools. This process does not cover Markush and generic structures. Contrary to the publicly available static IBM repository, SureChEMBL is updated daily, and, in addition to the web interface, it provides quarterly downloads of structures and compound-patent mapping files. The system currently contains more than 16 million compounds (with unique canonical SMILES) extracted from nearly 13 million patent documents.

With the growing number of automatically generated databases containing chemical structures derived from patents, it seems timely to explore ways to assess whether these databases are fit for purpose and to compare them in a fair and systematic manner.

### When is a patent chemistry database fit for purpose?

Not surprisingly, this question can only be responded to by another question: What is the specific purpose? The fact that in their simplest form patent chemistry databases create links between only two concepts reduces the number of possible use cases to two categories; one starting from chemical structures and the other starting from the patent document, as specified by its unique ID.

### Patent→compounds

For this generic use case, the expectation will be that for a given patent the database contains the correct structural representation of *all* chemical entities described in the patent (irrespective of where the entities occur in the patent document and the actual representation, e.g. chemical names, synonyms, structural depictions, Markush representations) with the link to the respective patent ID. As is immediately obvious to anyone who has worked with patents, to achieve this by means of manual curation is far from trivial but seems almost impossible when using automation. However, despite the realisation that this is very challenging, it is very likely that users will have extremely high expectations (even for automatically generated databases), which in turn makes it quite probable that users will view patent chemistry databases as not being fit for purpose for this specific task. Why are expectations so (unrealistically) high? For one, users will feel that (rightly or wrongly, depending on the number of patents) this task can be performed manually by one person in a reasonable time frame and maybe even more importantly, it is straightforward to establish how accurate and comprehensive the results are.

It is worth pointing out that although users will expect that *all* chemical entities in patents are correctly described in a patent chemistry database, it is very likely that for specific use cases users are looking for a way to identify relevant subsets of compounds, e.g. in patents that describe chemical syntheses they might want to be able to differentiate between starting materials, intermediates, and the final products. Similarly, if what is of interest is novel chemical matter, as claimed in patent documents, all other extracted chemistry, such as reagents and fragments, will not be seen as relevant.

### Compound→patents

Starting from a chemical structure, a user would want to be able to identify all patents in which the chemical entity corresponding to the chemical structure is referred to. Since there really is no manual ‘Plan B’ (i.e. a way to achieve this without a database) for this task, the user is more likely to accept that the automated workflow will not achieve 100 % coverage and that consequently some links between a chemical structure and a patent document will be missed. The crucial question is how many of such links will most likely be missed and how this varies across the different databases that are available. This information will allow the user to decide whether or not the level of risk for missing compound-patent links is acceptable and which database to use to minimise this risk.

Our results for an assessment of the two freely available patent chemistry databases SureChEMBL and IBM SIIP in the context of the two use cases patent**→**compounds and compound**→**patents are reported here.

## Results

### Use case 1: patent→compounds

In order to evaluate how reliable patent chemistry databases are for this type of use case, it is necessary to define a reference set of patents that contain chemical entities and to extract the chemical structures for these entities in a way that allows for them to be used as ‘gold standard’ for comparisons with patent chemistry databases. As far as a reference set of patents is concerned, we decided to take advantage of the work by Akhondi et al. [10], who have produced an Annotated Chemical Patent Corpus. However, since the annotation of the patent corpus was done through text mining only (images were removed from patents) and chemical names were not translated into chemical structures, we were still faced with the question of how to generate the list of chemical structures for the chemical entities in the patents. We decided to produce the reference list of chemical structures by querying the well-established, manually-curated CAS (Chemical Abstracts Service) Registry [11] through SciFinder [12]. Since we were only able to retrieve the chemical structures through the SciFinder web application for one patent at a time, due to the lack of a suitable API (Application Programming Interface) functionality, we restricted ourselves to the harmonised subset of 47 patents described by Akhondi et al. [10] and not the entire set of 200 patents. For the 47 patents covering the period 1980–2010 SDF files were exported from SciFinder with the chemical structures of all the molecules in the CAS Registry associated with these patents, as well as SDF files that only contained the structures of molecules that had the annotation “Biological Studies” in SciFinder. The rationale for the latter was that the molecules annotated with the term “Biological Studies” tend to be the molecules that are most relevant and that are the listed examples. Since we hypothesised that as part of the manual extraction of chemical information from patents these molecules might possibly get more attention than others, we wanted to be able to look at them separately. For one patent (US4874794, linked to WO9013287 in SciFinder), no chemical structures could be exported as SDF file.

First, we compared the results obtained with SciFinder with those from SureChEMBL. To perform the comparison, the workflow tool Pipeline Pilot from BIOVIA [13] was used. As input, the workflow used here takes an SDF file with the chemical structures from SciFinder and a text file with the SMILES from SureChEMBL. For both inputs canonicalised SMILES and InChI strings (standard InChI, version 1) [14] were generated for the largest fragment. For example, for metoprolol tartrate canonicalised SMILES and InChI strings would only be generated for metoprolol itself (with 38 non-hydrogen atoms, whilst the tartaric acid part of metoprolol tartrate only consists of 10 non-hydrogen atoms). The canonicalised SMILES and the InChI strings, respectively, were used to determine which molecules from SciFinder are also contained in the input file from SureChEMBL. If not specifically stated otherwise, the results below refer to the InChI-based comparison.

The total number of molecules derived from SciFinder for the remaining 46 patents was 2692. The average number of molecules per patent was 59, ranging from 2 to 277, which is in line with what Southan et al. [15] reported. From the 2692 molecules only 1588 (i.e. 59.0 %) were also found in the corresponding patent in SureChEMBL (cf. Additional file 1 for details). When the analysis was restricted to molecules with the annotation “Biological Studies” in SciFinder, the situation was broadly similar. For 35 of the 46 patents SciFinder contains molecules with the annotation “Biological Studies”. The total number is 950. Only 578 of these molecules were also found in SureChEMBL. This equates to 60.8 %, similar to what was found when looking at all molecules in a patent. When canonicalised SMILES were used for the comparison the overall results were very similar to the % values stated above. The observed overall differences were ≤0.5 %. When in addition to using the largest fragment only, compounds were further standardised by removing stereochemistry, the results improved significantly. SureChEMBL returned 64.9 % of the molecules in SciFinder when using these settings. With 67.1 % the percentage for the “Biological study” annotated subset is even higher.

**Fig. 1:**
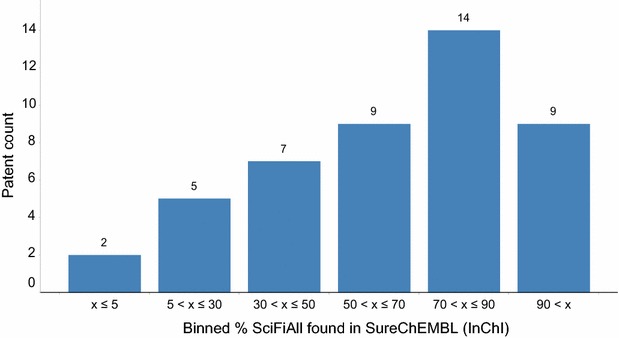
Distribution of SureChEMBL-SciFinder overlap. Number of patents where the percentage of hits from SciFinder that are also present in SureChEMBL is within a given range

As can be seen in Fig. 1, for seven patents no more than 30 % of the molecules in SciFinder were also found in SureChEMBL. We decided to investigate in more detail why the overlap for these patents was so small.

US4231938 was the only patent where none of the molecules in SciFinder was also found in SureChEMBL. However, it has to be said that for this patent SciFinder only contains two parent compounds (and one salt). The chemical structures of the compounds are only contained in the patent as depictions explaining why they are not in SureChEMBL. The publication year for this patent is 1980 but structures are only derived from images in SureChEMBL from 2007 onwards.

For the patent US4472305 only 1 out of 277 compounds from SciFinder was also found in SureChEMBL. In this patent hexapeptides are claimed. They are described using the three-letter codes for amino acids. These are not recognised by SureChEMBL as the necessary conversion of an amino acid sequence into chemical structure is not part of the SureChEMBL workflow.

For the patent WO2008129280 only 12 (19.4 %) of the 62 compounds in SciFinder were also found by SureChEMBL. 34 of these were compound names in a table, which was attached to the patent as an image, and SureChEMBL only recognizes 2D structures from images, not text from images. The other 16 compounds missing were not recognized as they are described as Markush structures.

The patent US4847265 has five associated compounds in SciFinder, only one of which was correctly extracted by SureChEMBL (20 %). From the missing matches, one compound was extracted with incorrect (missing) stereochemistry by SureChEMBL. The remaining three are in fact the same compound with different stereochemistries. They originated from an image, which was not resolved to structure, as the patent was published before 2007.

In patent US4738974, only one compound was returned by SureChEMBL out of the four in SciFinder (25 %). The three missing compounds are not explicitly present in either the text or in structural depictions; they are explained in the text as different salts of a compound. For example, there is a paragraph titled “Preparation of tetra-omeprazole titanium salt” which explains how it is prepared but does not explicitly state the name of the compound.

For the patent US6156756, SureChEMBL returned 22 out of the 81 compounds in SciFinder (27 %). Most of the missing compounds came from Markush structures; or SureChEMBL resolved them but with incorrect stereochemistry. In one case, the name of the compound was in the first line on the page, which had not been correctly recognized as text.

Lastly, in case of patent WO2006083612, SureChEMBL returned 19 out of the 68 compounds in SciFinder (28 %). In each of these cases the missing compounds came from Markush structures or images. Again, SureChEMBL by design does not resolve Markush structures, and images were also not resolved prior to 2007.

### How to find the needles in the haystack?

**Fig. 2:**
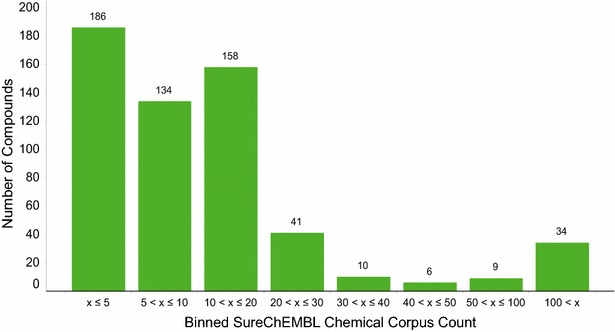
SureChEMBL Chemical Corpus Count of Compounds. Number of compounds from the set of 35 patents with the SciFinder annotation “Biological Studies” within eight ranges of SureChEMBL chemical corpus count

Considering that in SureChEMBL chemical entities are automatically extracted from patent documents (regardless of whether they are claimed compounds, reagents, synthetic intermediates etc.), it might not be surprising that the number of molecules associated with a patent in SureChEMBL tends to be much larger than the corresponding number in SciFinder. For the set of 46 patents used for this analysis only 12 % of compounds associated with the patents in SureChEMBL are also associated with these patents in SciFinder. For compounds annotated with “Biological Studies” in SciFinder the overlap is only 5 %. This prompts the question of how to identify the relevant compounds amongst what one could call artefacts or noise (e.g. reagents, intermediates, solvents, radicals, fragments, reference compounds, and existing marketed drugs). A SureChEMBL annotation that can help to address this challenge is the “chemical corpus count”. It is the count of the number of occasions a chemical structure has been observed within the whole currently available chemical corpus of SureChEMBL. Figure 2 shows how the compounds from the subset of 35 patents, for which in SciFinder compounds with the annotation “Biological Studies” can be found, are distributed across different ranges of chemical corpus count. As can be seen, approx. 90 % of the compounds have a chemical corpus count of less than or equal to 30. At the same time, only 16 % of all molecules found in SureChEMBL for the 35 patents have a chemical corpus count less than or equal to 30. Hence, applying a filter of chemical corpus count ≤30 enriches compounds that we consider here as being of greatest interest by a factor of 5. It is worth pointing out that, as expected, the majority of the molecules with the SciFinder annotation “Biological Studies” that have larger values for the chemical corpus count are, for example, marketed drugs or well known pharmacological tool compounds that are of minor relevance in the context of the use cases discussed here. The SureChEMBL output contains a number of further annotations (e.g. Ring Count, Molecular Weight, Log P, Radical, Med Chem Alert) that can also be used for filtering depending on the specific use case. Similarly, substructure searching is a viable option in cases where patents contain compounds that share a common structural entity. Examples of further data mining and cheminformatics methods applied to SureChEMBL compounds from patent documents are available as IPython Notebooks [16]. The notebooks aim to identify genuinely novel structures and series as claimed in a patent, along with methods to retrospectively flag key compounds [17, 18].

We also compared the results obtained with SciFinder with the IBM SIIP patent data.^1^ To perform this comparison, the same Pipeline Pilot workflow that was previously generated for the comparison with SureChEMBL was used. InChI strings were used to determine which molecules from SciFinder are also contained in the input file for IBM SIIP. It is worth noting that the total number of (non-unique) molecules for the 46 patents in IBM SIIP is 7027 (see Additional file 2), whereas SureChEMBL contains a total number of 12,801 compounds for the same patents.

From the 2692 molecules found in SciFinder for the 46 patents, only 1362 (i.e. 50.6 %) were also found in the corresponding patent in IBM SIIP (cf. Additional file 3 for details). When we restricted our analysis to molecules with the annotation “Biological Studies” in SciFinder, the situation was broadly similar. For 35 of the 46 patents SciFinder contains molecules with the annotation “Biological Studies”. Only 469 (out of 950) of these molecules were also found in IBM SIIP. This equates to 49.4 %, similar to what was found when we looked at all molecules in a patent.

**Fig. 3:**
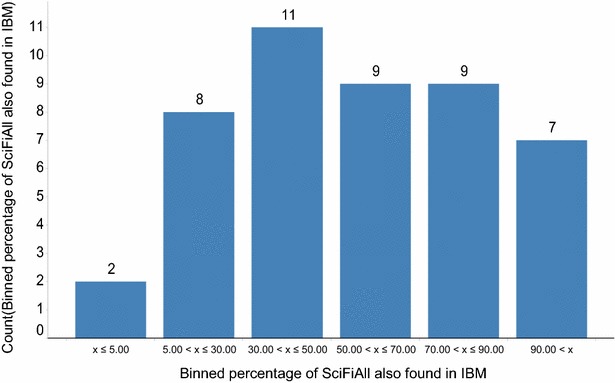
Distribution of IBM-SciFinder overlap. Number of patents where the percentage of hits from SciFinder that are also present in IBM SIIP is within a given range

As can be seen in Fig. 3, the number of patents for which no more than 30 % of the molecules in SciFinder were also found in IBM SIIP is slightly greater than was the case for SureChEMBL (10 versus 7, cf. Fig. 1). Also, for 35 % of the patents, IBM found over 70 % of the hits returned by SciFinder, whereas in SureChEMBL this was the case for 50 % of the patents. As before, we decided to investigate the patents with an overlap of 30 % or less in more detail.

As can be seen in Table 1, the two patents with the smallest overlap between SciFinder and IBM SIIP are the same as for SureChEMBL. The likely reasons why molecules have not been correctly identified have already been described above.

**Table 1:** List of patents where the percentage of compounds found in SureChEMBL or IBM is low

Patent number	Number of compounds in SciFinder	% SciFinder compounds in IBM SIIP	% SciFinder compounds in SureChEMBL
US4231938	2	0.0	0.0
US4472305	277	0.4	0.4
WO2009126123	169	5.3	*38.5*
EP1481667	47	14.9	*74.5*
WO2008129280	62	17.7	19.4
US4847265	5	20.0	20.0
US6323216	20	20.0	*40.0*
WO2006083612	68	23.5	27.9
US4738974	4	25.0	25.0
US4572909	60	28.3	*51.7*
US6156756	81	*32.1*	27.2

List of patents where the percentage of compounds in SciFinder that were also found in SureChEMBL or IBM SIIP is equal to or less than 30 %. Percentages above 30 % are marked in italics

For WO2009126123 the overlap between IBM SIIP and SciFinder is only 5.3 % compared to 38.5 % for SureChEMBL. With a factor of 7.2, this is the largest discrepancy that was observed for this set of patents. All molecules found by SureChEMBL have been identified from text and hence there is no obvious reason why they should not also be correctly identified by IBM SIIP.

For EP1481667, again there is a large difference (of a factor of 5) between IBM SIIP and SureChEMBL. All molecules in SureChEMBL have been identified from text and it is unclear why they have not been correctly identified by IBM SIIP.

For WO2008129280 the difference between IBM SIIP and SureChEMBL is minimal. SureChEMBL identified one compound that IBM SIIP missed. The compound in question is simply referred to as “aminophenol” in the text of the patent. Only in a table which appears to be an image it is specified that compound is 2-aminophenol, which is also what is captured in SciFinder. SureChEMBL provides two structures for the term “aminophenol”, i.e. 2-amino- and 4-aminophenol whereas IBM SIIP resolves the term to 4-aminophenol. Consequently, the 2-aminophenol from SciFinder could only be matched with the compound in SureChEMBL.

For US4847265 only one out of the five compounds that are in SciFinder have been correctly identified both by IBM SIIP and SureChEMBL.

For US6323216 SureChEMBL correctly identified four compounds that IBM SIIP did not retrieve correctly. One of the compounds is example 9 (“(R,S)-2-[1-azadicyclo[2.2.2]oct-3-yl]-2,3-dihydro-1H-pyrrolo[3,4-c]quinolin-1-one”). IBM SIIP extracted the radical “1-azadicyclo[2.2.2]oct-3-yl”, which suggests that instead of using the entire name of the compound for the name to structure conversion it only used part of it. We investigated whether it had been caused by the start of a new line in the patent document, but this was not the case.

For the patent WO2006083612, SureChEMBL had three additional compound matches with SciFinder when compared to IBM-SIIP. All three compounds were recognised from text.

For US4738974, both patent databases performed identically. Both of them correctly identifying 25 % of the compounds extracted by SciFinder.

Finally, in the case of patent US4572909, IBM only retrieved approx. 55 % of the compounds in SciFinder that were retrieved by SureChEMBL (i.e. 17 vs. 31 compounds). On closer inspection it appears that 11 of the 14 IBM SIIP compounds that were not matched with compounds from SciFinder contain an imine substructure whereas the compounds in SureChEMBL and SciFinder (in accordance with the chemical drawings in the patent) contained the corresponding enamine substructure. The standard InChIs we used are different for these tautomers and hence compounds were not matched.^2^

**Fig. 4:**
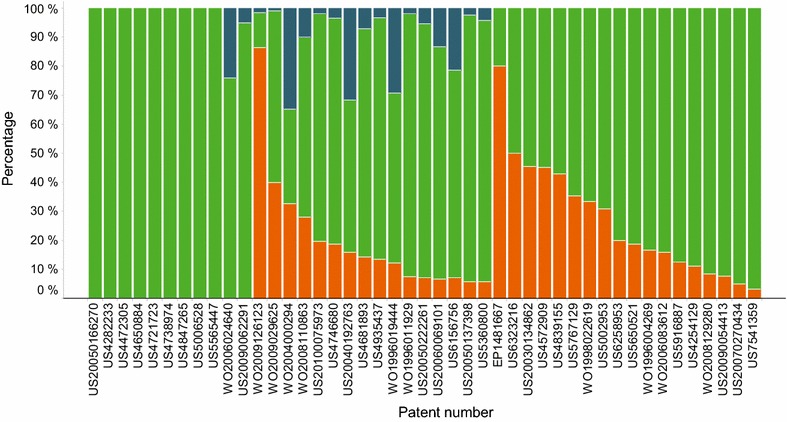
Distribution of compounds found by one or both of the two automatically curated databases. Percentage of compounds that are in SciFinder for the set of 45 patents and are only in SureChEMBL (*orange*), IBM SIIP (*blue*), or both (*green*). US4231938 is not included since none of compounds in SciFinder have been found in either SureChEMBL or IBM SIIP (cf. Table 1). The results used to generate this visualisation can be found in Additional file 3

In order to better understand how much overlap there is between compounds that are in SciFinder as well as in SureChEMBL and/or IBM SIIP for the set of 46 patents, the relevant information is shown in Fig. 4. As can be seen, there are 9 patents where every compound in SciFinder that was identified by one of the data sources was also found by the other. For one patent, none of the compounds in SciFinder have been found in either SureChEMBL or IBM SIIP. For two of the patents IBM SIIP found 32 additional compounds that are not in SureChEMBL, whereas SureChEMBL did not find any compound that is not also in IBM SIIP. For 16 patents SureChEMBL found compounds that IBM didn’t find and vice versa. Across these 16 patents SureChEMBL found 207 molecules that IBM didn’t find but IBM SIIP identified only 69 molecules that were missed by SureChEMBL. For the remaining 18 patents only SureChEMBL found compounds that were not identified by the other data source. In these 18 patents SureChEMBL found 120 additional compounds.

### Use case 2: compounds→patents

To study this use case, we assembled a list of compounds that appear in at least one patent. Our starting point was the Maybridge HitFinder collection of 14,400 compounds [19]. From this collection, we selected a subset of molecules with a heavy atom count of greater than 19 and a molecular weight of less than 500. The subset consists of 9274 compounds and was used as input for Reaxys [20], where an exact chemistry search was performed and hits were filtered based on document type to identify compounds that occur in patents. This resulted in a list of 660 compounds (with a unique standard InChI) with the associated patents that were found in Reaxys. For 114 of these compounds Reaxys only contained patents that are outside of the coverage of SureChEMBL as well as IBM SIIP (i.e. originating from patent authorities other than EPO/USPTO/WIPO or patents published before 1976). These compounds were excluded from the list. The remaining 543 compounds were divided into two subsets. The first one contains 452 unique compounds with at least one EP, US or WO patent (in Reaxys) from the period 1976 to 2010 (i.e. the patents that fall within the coverage of both SureChEMBL *and* IBM SIIP). After a manual quality check, 14 of these compounds were removed because they had incorrect or ambiguous structures. They were all complexes with three or more components. The resulting subset of 438 compounds was used for the comparison between SureChEMBL and IBM SIIP. Just to note, amongst the 438 are 99 compounds that contain two or three molecular components that are not covalently bound (e.g. salts). Before the patents were retrieved for the 438 compounds, the SMILES strings for those with nitro groups were standardized as we found that otherwise results are missed when retrieving structures from SureChEMBL. The second smaller subset contains 91 compounds with EP, US or WO patents (in Reaxys) that are all for the period 2011 to the present (i.e. patents that are within the coverage of SureChEMBL but not in IBM SIIP).

The first question we asked was how many of the 438 unique compounds are found in SureChEMBL and IBM SIIP, respectively. In an ideal setting, all compounds should be found in both sources since according to Reaxys at least one patent exists that falls within the coverage of both sources. We determined whether or not a compound was in IBM SIIP by using the UniChem API [8]. UniChem contains IBM SIIP compound IDs, which were retrieved using the “Get src_compound_ids from InChIKey” API call. For SureChEMBL, we retrieved all patents directly from the database.

**Fig. 5:**
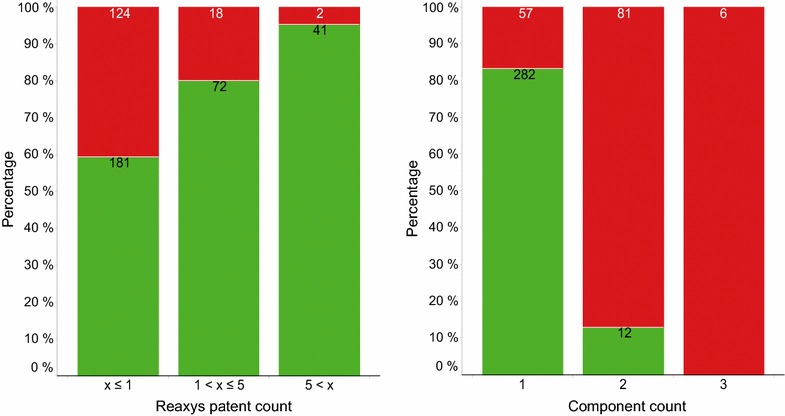
*Bar charts* showing the number of substances found in the databases. *Bar charts* with the number of substances found (*green*) or not found (*red*) in at least one of the two patent databases depending on the number of patents in Reaxys (*left*) and the number of components (*right*)

From the 438 compounds, a total of 294 compounds were found in at least one of the two sources. 231 compounds were found in both sources, 13 compounds were only found in IBM SIIP, and 50 compounds were only found in SureChEMBL. Hence, 55.7 % of the 438 compounds were found in IBM SIIP and 64.2 % were found in SureChEMBL. For the set of 91 compounds mentioned above (covering the period 2011 onwards) only 50 compounds (54.5 %) were found in SureChEMBL. To reiterate, at this point we only checked for the presence or absence of particular compounds in the two patent databases. Figure 5 (left) shows how the likelihood that a compound is found in at least one of the patent databases increases with the number of patents that were found for a compound (in Reaxys). For example, 59.3 % of compounds with only one patent in Reaxys were found whereas almost all molecules (95.3 %) with more than five patents in Reaxys are in either SureChEMBL or IBM SIIP (or in both). This is exactly what one would expect to observe. Figure 5 also shows (on the right) how the likelihood of a compound being found in either SureChEMBL or IBM SIIP seems to depend on the number of components it contains. Whereas 83.2 % of compounds with only one component were found, only 12.9 % of compounds with two components (e.g. salts) were found. None of the six compounds with three components were found in either SureChEMBL or IBM SIIP. This finding suggests (maybe not surprisingly) that it is more challenging to extract multi-component compounds correctly from patents than it is to extract individual molecules. Depending on whether or not this plays a role in relevant use cases will most likely have a significant effect on the result.

For the set of 438 compounds, 1740 unique compound-patent pairs were found in Reaxys (containing 1495 unique patent numbers). The second question we looked at for this use case was how many of the compound-patent pairs are found in SureChEMBL or IBM SIIP, respectively. Using the IBM SIIP compound IDs previously retrieved from UniChem, we downloaded files containing patent IDs from https://healthchem.almaden.ibm.com/ using the HTTP connector component in Pipeline Pilot. For SureChEMBL, we had already generated the list of patents for all compounds for the previous use case.

Before doing the comparison, the patent numbers originating from Reaxys had to be standardised. This was carried out in multiple steps. First of all, any ‘/’, ‘-‘characters were removed together with the kind code (last one or two characters, an uppercase letter and an optional number) where present. In the next step, the resulting patent numbers were converted to the format of the unique identifier referred to as SureChEMBL Patent Number (SCPN). The patent number format used for the comparison consisted of the patent office (two letters) and the number string, however, the SCPN format (PO-n-KK, where PO is the 2-digit patent office code, n is the patent number and KK is the 1- or 2-digit kind code) was kept for later use.

From the 1740 unique compound-patent pairs (found in Reaxys) 1233 (70.9 %) were found in at least one of the two patent chemistry databases. 871 of these 1233 pairs were found in SureChEMBL as well as in IBM SIIP. 1032 pairs (59.3 %) were found in IBM SIIP, 1072 pairs (61.6 %) were found in SureChEMBL. The matching was done based on the InChI of the compounds and the patent number (without the kind code). As discussed above (cf. Fig. 5), these results are heavily influenced by how often compounds occur in patents. For example, if one excludes compounds that are found in more than 10 patents (in Reaxys) the percentage of compounds found in at least one of the two patent chemistry databases drops from 70.9 to 54.1 % (i.e. 364 out of a total of 673 pairs).

To investigate the impact of deriving chemical structures from images, we split the 695 compound-patent pairs with WO patents in two groups depending on the year the WO patent was published. One group contains the 373 pairs with WO patents published before 2007 and the other group contained the 322 pairs with patents published in 2007 or later. Starting from 2007 the SureChEMBL workflow derives structures from images as well. In the first group, 54.4 % of the pairs are found in SureChEMBL whereas 62.1 % of the pairs in the second group are found. As expected, the processing of images seems to have improved the result. However, the change we observed here is smaller than one might have expected. A likely explanation could be that many of the images in the patent documents are in fact Markush structures.

Table 2 shows how the percentage of compound-patent pairs that were found in at least one database varies across patents from different patent authorities. The best result (74.6 %) was obtained for US patents. In contrast, for compound-patent pairs with WO patents or EP patents only approx. 67 % of the pairs were found. This finding prompted us to extend the use case to a scenario where we look for the presence of compounds in at least one of the patents that belong to the same patent family as opposed to a specific patent.

**Table 2:** Results from the search for 1740 compound-patent pairs in SureChEMBL (SC) and IBM SIIP (IBM)

Country code	Total	Not found	Total found	% Found	IBM only	SC only	Both
EP	172	57	115	66.9	27	21	67
US	873	222	651	74.6	70	102	479
WO	695	228	467	67.2	64	78	325
All	1740	507	1233	70.9	161	201	871

For the 1740 compound-patent pairs from Reaxys we retrieved all family members from the SureChEMBL database using the previously generated SCPN numbers. For this use case, only patent family members from US, EP or WO offices were retained. Using these alternative patent numbers along with the originals coming from Reaxys, SureChEMBL returned 1156 matches out of the 1740 patents (66.4 %). The output of IBM was also compared to the patent family list. This resulted in 1132 matches with the Reaxys patents (65.1 %). With the addition of family members, the overlap between the two data sources and Reaxys did not increase significantly: 1072 to 1156 (7.8 %) and 1032 to 1132 (9.7 %) matches for SureChEMBL and IBM SIIP, respectively.

## Discussion

Patent chemistry content has traditionally only been offered by commercial vendors. This has started to change in the last few years, with the release of public domain resources, which complement the already established bioactivity databases such as ChEMBL. The aim of this study was to explore ways to assess the reliability of freely available chemistry databases generated by automated, large scale extraction of chemical structures from patents. The latter comprise a notoriously challenging corpus for text mining, due to obfuscation, ambiguity and optical character recognition (OCR) conversion errors during digitalisation. Currently, information on the reliability of these automatically generated databases is not available in the public domain which makes it challenging to judge what these databases can and cannot be used for. A factor contributing to the scarcity of information related to the reliability of such databases is the fact that it proves to be very challenging to assess this quality in a meaningful and reliable way. One key contributing factor is the fact that such an assessment only becomes practically feasible if access to a database that can be used as ‘gold standard’ is available. Even then, access alone is not sufficient since specific requirements in terms of functionalities (e.g. the ability to perform searches with lists and suitable options to export the results) need to be satisfied. For the purpose of this study we chose manually-curated commercial chemistry databases as references, acknowledging that we don’t really have a good understanding of how complete or reliable they are. Even though it was not part of this study to assess this, we came across at least one example where no compounds were found in SciFinder but SureChEMBL, for example, contained the aliphatic alcohols mentioned in the claims section of the patent US4874794 (one of the patents from the Annotated Chemistry Patent Corpus).

When developing the workflows used in this study we made a series of further choices (e.g. which patents/compounds to choose for our use cases, inclusion of patent families, to use InChIs for the structural matching, how to format the patent IDs before matching them) that will all have impacted on the results we have obtained. When performing the matching of chemical structures we decided not to remove stereochemistry and not to include anything in our workflow that attempts to match tautomers (beyond the use of standard InChIs [14]) since it strongly depends on the specific use case if it makes sense to do so or not. Hence, it is only prudent to highlight that all these choices should be carefully taken into account when drawing conclusions from the results described here. In this context it is worth noting that this study is as much an attempt to establish workflows to assess the reliability of automatically generated patent chemistry databases as it is about the actual results.

The detailed analysis of the patents with the biggest discrepancies between SciFinder and SureChEMBL or IBM SIIP has shown that a variety of reasons contribute to molecules not being correctly extracted. Some of the reasons are difficult to address since they are inherent features of a patent (e.g. low image quality, typos and OCR conversion errors), others (e.g. Markush structures) offer opportunities for future developments. An overview of examples for reasons why molecules are not extracted correctly can be found in the Additional file 4.

In the patent**→**compounds use case only 63 % of the 2692 compounds found in SciFinder for the set of 46 patents were found in SureChEMBL, IBM SIIP or both. With 59 % compared to 51 %, SureChEMBL contains on average 8 % more of the compounds found in SciFinder than IBM SIIP for the chosen set of patents. For the biologically relevant patent compounds (as determined by the “Biological Studies” annotation in SciFinder), the situation is broadly similar, although with 11 % the gap between SureChEMBL and IBM SIIP is slightly bigger. The overlap between SureChEMBL and IBM SIIP for the compounds found in SciFinder is only 75 %. SureChEMBL found approximately three times as many compounds not found by IBM SIIP than vice versa (i.e. 327 compared with only 101, respectively). For the 46 patents, 12,801 and 7027 compounds were found in SureChEMBL and IBM SIIP, respectively, whereas for the same patents SciFinder only contains 2692 compounds. This finding prompts the question what the reasons for this substantial discrepancy might be. One likely explanation is that as part of the name-to-structure conversion in the SureChEMBL and IBM SIIP worflow all recognised chemical names and synonyms are converted to structure irrespective of their relevance whereas the annotation workflow used for SciFinder is more selective. This leads to a situation where, for example, a list of substituents in the claims section is converted to separate chemical entities like the methyl radical. The latter is amongst the chemical structures from SureChEMBL for 37 of the 46 patents. In agreement with the above, only 6 % of all the chemical structures for the 46 patents that are in SciFinder are found in more than one of the 46 patents whereas 27 % of all the chemical structures for the 46 patents that are in SureChEMBL or IBM (but not SciFinder) occur in more than one of the 46 patents. Also, approx. 80 % of the chemical structures in SureChEMBL or IBM SIPP that occur in more than 5 of the 46 patents have a heavy-atom count of 10 or less. Hence, it appears that a large percentage of the compounds from SureChEMBL and IBM SIPP might not be particularly relevant. These observations highlight that for automatically generated patent chemistry databases strategies need to be devised that allow for the most relevant compounds to be easily identified amongst all the compounds associated with a given patent in these databases. These could be, for example, filters on molecular weight, patent corpus count or other properties.

For the compounds→patents use case 56 % of the 438 compounds were found in IBM SIIP and 64 % were found in SureChEMBL. In line with our expectations we found that the likelihood that a given compound is found in IBM SIIP or SureChEMBL increases with the number of patents (in Reaxys) referring to this compound. Similarly, our results show that it can be challenging for an automated extraction of chemical structures to correctly extract compounds containing more than one non-covalently bound molecular component (e.g. salts). The results we obtained when searching for the presence of 1740 unique compound-patent pairs in the two patent chemistry databases were very similar with 62 and 59 % of the pairs found, respectively. Allowing matches in other patents from the same patent family only lead to a moderate improvement of around 6 and 5 %, respectively.

## Conclusions

In our comparison of two freely available, large scale, automatically generated patent chemistry databases with their manually curated commercial counterparts, 50–66 % of the relevant content from the latter was also found in the former. This information will hopefully help to manage expectations of users of patent chemistry databases of this type. The challenges we have encountered whilst attempting to perform benchmarking of patent chemistry databases that automatically extract chemical structures from patents highlights that more needs to be done to make this task easier. An already ongoing effort is to make SureChEMBL data accessible via the Open PHACTS API [21, 22]. More importantly, more adequate, preferably open access to relevant ‘gold standards’ is required. If the latter is not provided it will be difficult to decide where to focus development efforts in the future.

## Additional files

10.1186/s13321-015-0097-z Table with the results for the InChI- and SMILES-based comparison between molecules in SciFinder and SureChEMBL for the list of 46 patents.
10.1186/s13321-015-0097-z Table with the results for the InChI-based comparison between IBM SIIP and SciFinder for the list of 46 patents.
10.1186/s13321-015-0097-z Table with the total number of compounds in SciFinder for a given patent that are also in either SureChEMBL or IBM SIIP or both.
10.1186/s13321-015-0097-z Table with examples for reasons why molecules are not successfully extracted from patent documents.

## Abbreviations

API: application programming interface
CAS: Chemical Abstracts Service
EFPIA: European Federation of Pharmaceutical Industries and Associations
EPO: European Patent Office
GPCR: G protein-coupled receptor
IBM SIIP: IBM Strategic Intellectual Property Insight Platform
IMI: Innovative Medicines Initiative
NCBI: National Center for Biotechnology Information
NIH: National Institutes of Health
OCR: optical character recognition
USPTO: United States Patent and Trademark Office
WIPO: World Intellectual Property Organization

## Footnotes

1 The IBM SIIP patent data was downloaded from the National Center for Biotechnology Information (NCBI) ftp server (ftp://ftp.ncbi.nlm.nih.gov) where it is located in the directory/pubchem/Other/IBM. For the comparison with SciFinder we consolidated the relevant lines from the downloaded files for each of the patents into one delimited file per patent.

2 InChI currently recognises mobile protons, however it does not support several other ways of tautomeric H migration. In particular, these are keto-enol and long-range tautomerisms [23].

## References

[1] Lowe D, Sayle R Chemistry and reactions from non-US patents. http://www.slideshare.net/NextMoveSoftware/chemistry-and-reactions-from-non-us-patents

[2] The ChEMBL-og. http://chembl.blogspot.co.uk/2012/05/how-far-behind-patent-literature-is.html

[3] Bregonje M (2005). Patents: a unique source for scientific technical information in chemistry related industry?. World Pat Inf.

[4] IBM Strategic IP Insight Platform (IBM SIIP). http://www-935.ibm.com/services/us/gbs/bao/siip/

[5] IBM press release. http://www-03.ibm.com/press/us/en/pressrelease/36180.wss

[6] Complex Work Unit Pilot Program. http://www.uspto.gov/patent/initiatives/complex-work-unit-pilot-program

[7] Bolton E, Wang Y, Thiessen PA, Bryant SH (2008) PubChem: integrated platform of small molecules and biological activities. In: Wheeler R, Spellmeyer D (eds) Annual Reports in Computational Chemistry, vol 4. Elsevier, Oxford, p 217

[8] Chambers J, Davies M, Gaulton A, Hersey A, Velankar S, Petryszak R, Hastings J, Bellis L, McGlinchey S, Overington JP (2013). UniChem: a unified chemical structure cross-referencing and identifier tracking system. J Cheminf.

[9] EMBL-EBI press release. http://www.ebi.ac.uk/about/news/press-releases/SureChEMBL

[10] Akhondi SA, Klenner AG, Tyrchan C, Manchala AK, Boppana K, Lowe D, Zimmermann M, Jagarlapudi SARP, Sayle R, Kors JA, Muresan S (2014). Annotated chemical patent corpus: a gold standard for text mining. PLoS One.

[11] CAS REGISTRY. The gold standard for chemical substance information. http://www.cas.org/content/chemical-substances

[12] SciFinder. http://www.cas.org/products/scifinder

[13] BIOVIA. Pipeline pilot overview. http://accelrys.com/products/pipeline-pilot/

[14] Heller S, McNaught A, Pletnev I, Stein S, Tchekhovskoi D (2015). InChI, the IUPAC International Chemical Identifier. J Cheminf.

[15] Southan C, Varkonyi P, Boppana K, Jagarlapudi SARP, Muresan S (2013). Tracking 20 Years of compound-to-target output from literature and patents. PLoS One.

[16] Papadatos G SureChEMBL IPython Notebook SureChEMBL Tutorial 2. https://www.github.com/chembl/mychembl/blob/master/ipython_notebooks/12_myChEMBL_SureChEMBL_tutorial_2.ipynb

[17] Hattori K, Wakabayashi H, Tamaki K (2008). Predicting key example compounds in competitors’ patent applications using structural information alone. J Chem Inf Model.

[18] Tyrchan C, Boström J, Giordanetto F, Winter J, Muresan S (2012). Exploiting structural information in patent specifications for key compound prediction. J Chem Inf Mod.

[19] Maybridge HitFinder Collection. http://www.maybridge.com/portal/alias__Rainbow/lang__en/tabID__229/DesktopDefault.aspx

[20] Reaxys. http://www.elsevier.com/online-tools/reaxys

[21] Open PHACTS. http://www.openphacts.org

[22] Open PHACTS API. http://dev.openphacts.org

[23] InChI Trust Technical FAQ. http://www.inchi-trust.org/technical-faq/#6.4

