# Supplementary material for: Managing expectations: assessment of chemistry databases generated by automated extraction of chemical structures from patents
Source: J Cheminform. 2015 Oct 6;7:49. doi: 10.1186/s13321-015-0097-z (PMC4594083; doi:10.1186/s13321-015-0097-z)
Supplement: Supplementary file 4 — 10.1186/s13321-015-0097-z Table with examples for reasons why molecules are not successfully extracted from patent documents. [file 13321_2015_97_MOESM4_ESM.pdf]

Examples for reasons why molecules are not successfully extracted from patent documents

		**format in patent document:**	
		**image**	**text**
**molecular structures described as:**	**structural depiction**	Image-to-structure conversion<br>• is not part of the workflow<br>• fails due to bad image quality, low image resolution, or complexity of the structural depiction (e.g. Markush structures)	The structural 'encoding' that is being used is not recognised (e.g. 3-letter amino acid codes for peptides or SMILES)
	**chemical name**	Chemical names are not extracted from images. For example, tables containing chemical names might be images	The name-to-structure conversion can fail due to<br>• typos in patents<br>• OCR errors<br>• parsing errors leading to partial names being used as input for the conversion (e.g. names for R-groups, part of a name due to a line break)<br>• chemical names recognition doesn't distinguish between R-groups and molecules (resulting in radicals, for example)<br>• use of non-systematic names leads to failure of the name-to-structure conversion if the names are not in the dictionary
